# Implementation of a ventilator care bundle to reduce the incidence of ventilator acquired pneumonia

**DOI:** 10.1186/1753-6561-5-S6-P71

**Published:** 2011-06-29

**Authors:** AK Mandal, A Sharma

**Affiliations:** 1 Pulmonary Medicine, Fortis Hospital, Mohali, India; 2 Infection control, Fortis Hospital, Mohali, India

## Introduction / objectives

Ventilator acquired pneumonia (VAP) is a nosocomial infection that occurs in patients receiving mechanical ventilation for at least 48 hrs. We observed a high rate of VAP of 18.3 per 1000 ventilator days in 2008 and 8.6 per thousand ventilatordays in 2009 in one of our intensive care units at Fortis Hsopital.Reduction of VAP through the implemetation of Ventilator care bundles was taken as one of the quality improvement initiative for the unit.

## Methods

The study was divided into preintervention, intervention and postintervention phases. Data was collected on the VAP rates, hand hygiene and ventilator care bundle practices of the team during preintervention and postintervention phase through knowledge surveys and observational surveys of the team. A workshop on care bundles supported by introduction of an insertion and maintainence tool for Ventilators and oral care were the interventions adopted.

## Results

The surveillance of ventilator bundle showed an improvement of 64% in the postintervention phase as compared to zero percent in the pre-intervention phaase. The VAP rates in 2010 after the introduction of the bundle towards the end of 2009 were observed to be significantly lower than those in 2008 and 2009. The average (mean) VAP rates over 12 months dropped from 8.6 per 1000 ventilator days in 2009 to 2.1 per 1000 ventilator days in 2010 over a span of one year.

## Conclusion

Implementation of ventilator care bundle resulted in decrease in VAP in the unit over a period of one year in 2010. A lot of other supporting activities like oral care, cleaning and disinfection of ventilator parts were improved uponi simultaneously and it would have also played a role in the reduced VAP rates.

## Disclosure of interest

None declared.

